# Safety and effectiveness of granulocyte and monocyte adsorptive apheresis in patients with inflammatory bowel disease in special situations: a multicentre cohort study

**DOI:** 10.1186/s12876-019-1110-1

**Published:** 2019-11-21

**Authors:** Satoshi Motoya, Hiroki Tanaka, Tomoyoshi Shibuya, Taro Osada, Takayuki Yamamoto, Hitoshi Hongo, Chiemi Mizuno, Daisuke Saito, Nobuo Aoyama, Toshihisa Kobayashi, Hiroaki Ito, Satoshi Tanida, Masanori Nojima, Seiichiro Kokuma, Eiji Hosoi

**Affiliations:** 1 IBD Center, Sapporo Kosei General Hospital, Kita-3, Higashi-8, Chuo-ku, Sapporo, Hokkaido 060-0033 Japan; 2 Department of Gastroenterology, Juntendo University School of Medicine, 2-1-1 Hongo, Bunkyo-ku, 113-8431 Tokyo Japan; 3 Department of Gastroenterology, Juntendo University Urayasu Hospital, 2-1-1 Tomioka, Urayasu, 279-0021 Chiba Japan; 4 Department of Surgery & Inflammatory Bowel Disease Center, Yokkaichi Hazu Medical Center, 10-8 Hazuyamacho, Yokkaichi, 510-0016 Mie Japan; 5 Fujita Gastroenterological Hospital, 17-36 Matsubaracho, Takatsuki, 569-0086 Osaka Japan; 6 Department of Gastroenterology and Hepatology, Saiseikai Suita Hospital, 1-2 Kawazonocho, Suita, 564-0013 Osaka Japan; 7 The Third Department of Internal Medicine, Kyorin University School of Medicine, 6-20-2 Shinkawa, Mitaka, 181-8611 Tokyo Japan; 8 Gastrointestinal Endoscopy and IBD Center, Aoyama Medical Clinic, 3-3-19 Tamondori, Chuo-ku, Kobe, 650-0015 Hyogo Japan; 9 Department of Gastroenterology, Hakodate Goryoukaku Hospital, 38-3 Goryokakucho, Hakodate, 040-8611 Hokkaido Japan; 10 Kinshukai Infusion Clinic, 3-1 Ofukacho, Kita-ku, Osaka, 530-0011 Osaka Japan; 11 Department of Gastroenterology and Metabolism, Nagoya City University Graduate School of Medical Sciences, 1 Kawasumi, Mizuho-cho, Mizuho-ku, Nagoya, 467-8601 Aichi Japan; 12 Center for Translational Research, The Institute of Medical Science Hospital, The University of Tokyo, 4-6-1 Shirokanedai, Minato-ku, 108-8639 Tokyo Japan; 13 Medical affairs, JIMRO Co., Ltd, 351-1 Nishiyokote, Takasaki, 370-0021 Japan

**Keywords:** Inflammatory bowel disease, Ulcerative colitis, Crohn’s disease, Granulocyte and monocyte adsorptive apheresis, GMA, Elderly patient, Paediatric patient, Immunosuppressant, Anaemia, Special situation

## Abstract

**Background:**

The available information on granulocyte and monocyte adsorptive apheresis (GMA) in patients with inflammatory bowel disease (IBD) under special situations remains unclear. We conducted a retrospective, multicentre cohort study to evaluate the safety and effectiveness of GMA in patients with IBD under special situations.

**Methods:**

This study included patients with ulcerative colitis (UC) or Crohn’s disease who had at least one special situation feature and who had received GMA between November 2013 and March 2017. The incidence of adverse events (AEs) was compared in relation to the special situation, and patient background factors related to an AE were identified. For patients with UC, clinical remission was defined as a partial Mayo score of ≤2.

**Results:**

A total of 437 patients were included in this study. The incidence of AEs among the elderly patients (11.2%) was similar in all patients (11.4%), whereas the incidences of AEs in patients on multiple immunosuppressant medications (15.2%), patients with anaemia (18.1%) and paediatric/adolescent patients (18.9%) were higher than that in all patients (11.4%). In multivariate analysis, anaemia and concomitant immunosuppressant medications were independently associated with the incidence of AEs. Clinical remission was achieved in 46.4% of the patients with UC.

**Conclusions:**

The incidence of AEs in the elderly patients was not higher than that in all patients, whereas the incidence of AE was higher in patients with anaemia and those on multiple immunosuppressant medications than that in all patients. GMA is a safe treatment option in elderly patients with IBD.

## Background

Inflammatory bowel disease (IBD), which includes ulcerative colitis (UC) and Crohn’s disease (CD), is an idiopathic, chronic, inflammatory intestinal disorder [1, 2]. Historically, IBD was known as a disease of the Western countries, but recently, the number of patients with IBD has been consistently growing in Asia and the Middle East [3]. The increase in the prevalence of IBD includes paediatrics [4], elderly [5] and other smaller sub-groups in ‘special situations’ [6, 7]. Accordingly, the guidelines for the treatment of elderly, paediatric, pregnant patients with IBD and patients with IBD in other special situations were recently published by the European Crohn’s and Colitis Organisation (ECCO) [6–10].

Currently, various pharmacological preparations including corticosteroids, thiopurines, anti-tumour necrosis factor (TNF) agents, anti-IL12/23, or anti-α4β7 integrin have become available for the treatment of patient with IBD. However, immunosuppressant medication is known to have some limitations, which is more serious in elderly and paediatric patients with IBD. It is known that the risk of opportunistic infection increases in patients with IBD on concomitant immunosuppressant medications and that it is even higher in those aged > 50 years [11, 12]. Especially, corticosteroids increase the risk of osteoporosis, hypertension, hypokalaemia and serious infections in elderly and are associated with growth impairment [13–15] and delayed puberty adolescent patients and adolescent patients with IBD [16]. Similarly, thiopurines in elderly patients with IBD reportedly carry an increased risk of non-melanoma skin cancer, lymphoproliferative disorder [17, 18] and opportunistic infections [19] when compared with younger patients with IBD. Furthermore, it has been reported that elderly patients with IBD treated with an anti-TNF agents such as infliximab, adalimumab or certolizumab pegol experience a higher incidence of severe adverse event (AE) together with treatment discontinuation as compared with younger patients [20, 21]. The bottom line derived from these observations is that extra attention is warranted to avoid increased incidences of AE when prescribing immunosuppressant medications to patients with IBD under special situations.

Granulocyte and monocyte adsorptive apheresis (GMA) is a non-pharmacological, extracorporeal therapy for IBD and pustular psoriasis, which is administered by applying the Adacolumn® (JIMRO Takasaki Japan) [22–24]. GMA selectively depletes elevated granulocytes and monocytes from the patients’ circulation system, but spares most of the lymphocytes [22]. GMA has been approved in Japan since October 1999 as an effective and safe remission induction therapy for active patients with IBD. In patients with IBD, during each of the active phase, each patient receives one or more GMA sessions per week, up to a maximum of 10, or 11 sessions in patients with fulminant UC [25]. In a post-marketing surveillance study reported by Hibi, et al. [26] that involved 656 patients with UC, the overall remission rate was 71.1%, with an AE rate of 7.7%. Both in patients with UC and CD, open-label, prospective, randomised, multicentre trials were conducted to compare two GMA sessions per week [intensive GMA group], with one GMA session per week (weekly GMA group) [27, 28]. The time to remission was significantly shorter in the intensive GMA group than in the weekly GMA group, both in UC (14.9 days vs 28.1 days; *P* <  0.0001) and CD (21.7 days vs 35.4 days; *P* = 0.0373) trials. In the UC trial, the remission rate was also significantly higher in the intensive GMA group than in the weekly GMA group (71% vs 54.0%; *P* = 0.029). No device-related serious AEs or unexpected event was observed in both the trials [27, 28].

In addition, several studies from Japan and European countries have been reported on the therapeutic effectiveness and safety of GMA in patients with UC [29–34], CD [31, 35–37] and generalised pustular psoriasis [38]. However, available information on the effectiveness and safety of GMA in patients with IBD under special situations [6, 7] remains unclear because elderly, paediatrics and other sub-groups with comorbidities are generally not included in routine clinical trials. Therefore, we conducted a post-marketing surveillance study referred to as the ‘Post-marketing surveillance study on the safety and response of GMA treatment in patients with Crohn’s disease or ulcerative colitis with at least one special situation who received Adacolumn®’ (PARTICULAR). The aim of the present study was to evaluate the safety and effectiveness of GMA in patients with IBD under special situations.

## Methods

### Patients

This retrospective, multicentre cohort study included only those patients with UC or CD who had at least one special situation feature and who had received GMA therapy in the medical institutions of Japan between November 2013 and March 2017.

Patients with special situation [6, 7] were defined as those with at least one of the following features: 1) elderly (age ≥ 65 years); 2) paediatric/adolescent (age ≤ 18 years); 3) pregnant or lactating mothers, 4) patients with renal failure (serum creatinine ≥1.3 mg/dL for male patients and ≥ 1.2 mg/dL for female patients) or patients who needed regular haemodialysis; 5) patients with liver malfunction based on the serum aspartate aminotransferase (AST) or alanine aminotransferase (ALT) level being > 2 times the normal laboratory levels (or with confirmed liver cirrhosis); 6) patients with anaemia, with haemoglobin (Hb) level < 10 g/dL; 7) patients with impaired cardiac functions; 8) patients with past or present malignancy; 9) patients on multiple immunosuppressant medications, including corticosteroid; thiopurines, including azathioprine and 6-mercaptopurine; calcineurin inhibitors, such as tacrolimus and cyclosporine; methotrexate or anti-TNF agents, including infliximab and adalimumab, at baseline or at the 5th GMA session; 10) patients who received GMA retreatment (patients who had receive GMA therapy in the past and received the GMA therapy again following a clinical relapse) and 11) any patient who was judged by the physician to meet the criteria of a special situation [6, 7].

### GMA therapy

GMA therapy was performed using the Adacolumn®, as previously described [2, 23]. Patients received one or more GMA sessions/week, up to 10 sessions (or 11 sessions in fulminant UC) as per the national health reimbursement scheme in Japan [25]. The duration of one GMA session was 60 min at a blood flow rate of 30 mL/min [22, 23]. Blood access was via the antecubital vein in one arm and from the Adacolumn® outflow, the blood returned to the patient via a venipuncture in the antecubital vein in the contralateral arm. In general, prior to each GMA session, the extracorporeal (apheresis) system was primed by running 1 l of physiologic saline followed by another 1 l of saline containing an anti-coagulant to complete the priming of the Adacolumn® system. During GMA, heparin (including low-molecular weight heparin) or nafamostat mesylate was used as the anti-coagulant for continuous infusion during the 60 min GMA session. A typical dose of heparin was 4000 U for system priming and 2000 U for continuous infusion. The dose of nafamostat mesylate was 20 mg for system priming and 30 mg for continuous infusion.

### Data collection

The observation time interval was from baseline (within 2 weeks before the 1st GMA session) to the final assessment time (within 1 month after the 10th or 11th GMA sessions in patients with UC and the 10th session in patients with CD). In patients who had to discontinue GMA, the final assessment time was within 1 month of the last GMA session. At baseline, the patients were reviewed for the following demographics: age, gender, duration of disease, extent of intestinal involvement (proctitis, left-sided, extensive, ileal, colonic and ileocolonic) according to the Montreal classification of IBD [39]. All AEs were recorded during the observation time interval. In addition, feasibility problems (FPs) during the operation of the GMA column, including difficulty in achieving blood access, technical problems related to the system operation, venous pressure fluctuation and coagulation in the apheresis system lines were recorded. The reason for the patient to discontinue GMA due to AE, FP, physician’s decision or patient’s own will was also recorded. Furthermore, the number of GMA sessions and the type of anti-coagulant (heparin, low-molecular weight heparin or nafamostat mesylate) that each patient had received at the first GMA session was also recorded.

In patients with UC, the disease activity was evaluated by applying the partial Mayo (pMayo) score [40]. The pMayo score comprises three non-invasive components: stool frequency, rectal bleeding and physician’s global assessment, but it is without an endoscopic sub-score [40]. Patients with CD were evaluated according to the methods of de Dombal and Softley [41], and Myren, et al. [42], which factor the International Organization for the Study of IBD (IOIBD) guidelines pertained to CD. The pMayo and IOIBD scores were recorded at baseline and at the final assessment time. The dosage and the number of concomitant medications (5-aminosalicylate, corticosteroid, azathioprine, 6-mercaptopurine, tacrolimus, cyclosporine, infliximab, adalimumab or methotrexate), together with the laboratory data, including C-reactive protein (CRP), white blood cell count, platelet count, haematocrit and Hb were recorded at baseline, at the 5th GMA session and at the final assessment time. Data were compiled by the institutions and then provided to JIMRO.

### Assessment of GMA safety

Any patient who received at least one GMA session was eligible for the assessment of GMA safety. Any AE was coded according to the Medical Dictionary for Regulatory Activities (MedDRA/J version 15.1). Similarly, the serious AE (sAE) was determined in accordance with the classification criteria for seriousness of adverse reactions to pharmaceuticals [43]. Any AE for which the causality of GMA could not be ruled out was classified as adverse device effect (ADE) by the participating physician or another registered investigator.

Essentially, we were interested in evaluating the following: 1) the incidence of AE, sAE, ADE and FP; 2) comparison of the incidences of AE, ADE and FP between the major special situations with an adequate number of patients and 3) identification of patient background factors that relate to an AE using multivariate analysis.

### Assessment of GMA effectiveness

Patients with UC who started the GMA therapy with the pMayo score > 2 and patients with CD who started with the IOIBD score ≥ 2 or CRP > 0.3 mg/dL at baseline were considered eligible for the assessment of GMA effectiveness. Patients who were receiving an anti-TNF agent, calcineurin inhibitor or methotrexate at baseline were excluded from the assessment of effectiveness for the purpose of evaluating the full effectiveness of GMA in special situation. Likewise, patients with missing pMayo score, IOIBD score or unknown CR*P* value were excluded from the assessment of effectiveness. Patients receiving multiple immunosuppressant medications were sub-grouped under ‘patients on corticosteroids with azathioprine or 6-mercaptopurine’ in the assessment of effectiveness.

For patients with UC, clinical remission was defined as pMayo score ≤ 2, with no individual sub-score exceeding 1 at the final assessment time, while the clinical response was defined as a decrease in the pMayo score by ≥2 points and by ≥30% decrease relative to that at baseline plus all 3 sub-scores ≤1 at the final assessment time [44, 45]. For patients with CD, clinical remission was defined as an IOIBD score of 0 or 1 with CRP value of ≤0.3 mg/dL at the final assessment time, while clinical response was defined as ≥2 points decrease in the IOIBD score relative to that at baseline. Patients who received additional medications or increased dose of a concomitant medication such as corticosteroids, thiopurines, calcineurin inhibitors, anti-TNF agents or methotrexate during the course of GMA therapy because of unremitting IBD were considered as non-responders to GMA.

Furthermore, to assess the corticosteroid-sparing effect of GMA, we compared the corticosteroid dose at baseline with the dose at the final assessment time.

### Statistical analysis

Numerical data are presented as the median (interquartile range [IQR]) for continuous variables, while categorical variables are presented in the form of absolute numbers and percentages. The percent of AE, sAE, ADE and FP were calculated by using the formula: the number of patients experiencing at least one AE/total number of patients in the safety assessment × 100. 95% confidence intervals (CIs) for a fraction of patients were calculated by the Clopper-Pearson exact method. Furthermore, analyses of the dose of corticosteroid and the pMayo scores at baseline relative to the final assessment time was made by applying the Wilcoxon signed-rank test. To assess the factors that potentially affect GMA safety in patients with special situations, an adequate number of patients with major special situations were factored for analysis. Major special situation sub-groups and the presumed risk factors (age, gender, type of IBD, duration of disease, corticosteroid and the number of concomitant immunosuppressant medications) were considered as variables in the multivariate analyses, undertaken by applying a logistic regression model with a backward elimination method (variables with *P* > 0.2 were excluded). Odds ratios (OR) with 95% CIs were calculated for selected variables. The statistical significance level was set as *P* <  0.05 (2-sided test). All statistical analyses were performed using SPSS software, version 23.0 (SPSS, Chicago, IL, USA).

## Results

### Patients’ demographic variables

**Fig. 1:**
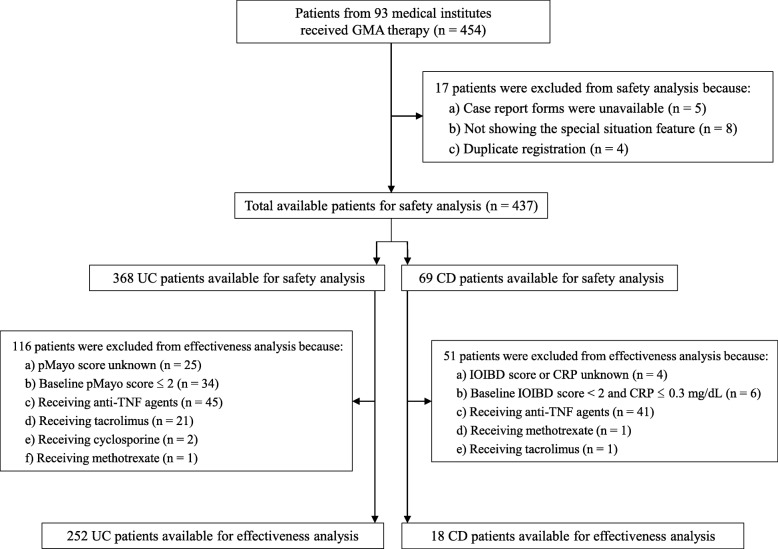
Patient selection. *GMA* granulocyte and monocyte adsorptive apheresis, *UC* ulcerative colitis, *CD* Crohn’s disease, *pMayo* partial Mayo, *IOIBD* International Organization for the Study of Inflammatory Bowel Diseases, *TNF* tumour necrosis factor, *CRP* C-reactive protein

Overall, 454 patients with IBD from 93 institutions were registered in the present study. Patient selection is summarised in Fig. 1. Among the 454 patients, 17 were excluded from the final safety or effectiveness assessment. The reasons for exclusions included case report forms not available (*n* = 5), no special situation feature (*n* = 8) and duplicate registration (*n* = 4). Additional file 1: Table S1 shows the baseline demographic variables of all patients in the present study. Among the 437 available patients, 368 had UC and 69 had CD. Table 1 illustrates the patients’ baseline demographic variables for inclusion after meeting the eligibility factors defined by the special situation. The major special situation sub-groups with an adequate number of patients included the following 5 groups: patients who received GMA retreatment (*n* = 131), those on multiple immunosuppressant medications (*n* = 125), elderly patients (n = 125), patients with anaemia (*n* = 105) and paediatric/adolescent patients (*n* = 53). Among the included patients, some patients showed more than one special situation features.

**Table 1:** Summary of the safety assessment in each sub-group within the patients in special situations

	Number of patients			Number and (%) of patients							
	Total	UC	CD	AE		sAE		ADE		FP	
Patients eligible for safety analysis	437	368	69	50	(11.4%)	16	(3.7%)	11	(2.5%)	71	(16.2%)
Patients who received GMA retreatment	131	109	22	13	(9.9%)	3	(2.3%)	3	(2.3%)	11	(8.4%)
Patients on multiple immunosuppressant medications	125	95	30	19	(15.2%)	5	(4.0%)	5	(4.0%)	23	(18.4%)
Elderly patients (≥ 65 years)	125	118	7	14	(11.2%)	7	(5.6%)	0		22	(17.6%)
Anaemic patients (haemoglobin < 10 g/dL)	105	89	16	19	(18.1%)	7	(6.7%)	4	(3.8%)	22	(21.0%)
Paediatric/adolescent patients (≤ 18 years)	53	40	13	10	(18.9%)	4	(7.5%)	3	(5.7%)	11	(20.8%)
Patients with diabetes mellitus	23	20	3	4	(17.4%)	1	(4.3%)	0		4	(17.4%)
Patients with ischaemic heart disease or arrhythmia	20	19	1	4	(20.0%)	2	(10.0%)	0		4	(20.0%)
Patients with viral hepatitis	19	18	1	3	(15.8%)	2	(10.5%)	0		6	(31.6%)
Patients with past or current malignancy	19	16	3	1	(5.3%)	0		0		3	(15.8%)
Patients with arrhythmia	16	14	2	2	(12.5%)	1	(6.3%)	0		1	(6.3%)
Patients with hypertension	16	16	0	2	(12.5%)	0		0		2	(12.5%)
Patients with liver disorder	15	14	1	5	(33.3%)	4	(26.7%)	0		1	(6.7%)
Patients with intestinal fistula	14	0	14	2	(14.3%)	0		1	(7.1%)	2	(14.3%)
Patients infected by cytomegalovirus	14	14	0	2	(14.3%)	1	(7.1%)	0		2	(14.3%)
Patients with renal disorder	13	11	2	1	(7.7%)	1	(7.7%)	0		1	(7.7%)
Pregnant or lactating mothers	12	12	0	0		0		0		1	(8.3%)
Patients with pyoderma gangrenous	9	7	2	0		0		0		1	(11.1%)
Patients with dyslipidemia	9	9	0	1	(11.1%)	0		0		0	
Patients refractory or intolerant to biologics	8	5	3	0		0		0		1	(12.5%)
Patients with erythema nodosum	8	8	0	0		0		0		1	(12.5%)
Patients intolerant to 5-aminosalicylates	8	7	1	0		0		0		2	(25.0%)
Patients with CD receiving GMA monotherapy	8	0	8	1	(12.5%)	1	(12.5%)	0		2	(25.0%)
Patients with psychiatric disorders	6	6	0	0		0		0		3	(50.0%)
Patients with primary sclerosing cholangitis	4	4	0	0		0		0		0	
Patients allergic to anticoagulants	4	4	0	3	(75.0%)	0		2	(50.0%)	3	(75.0%)
Patients with intestinal stenosis	3	0	3	1	(33.3%)	0		1	(33.3%)	1	(33.3%)
Others	12	11	1	3	(13.6%)	1	(8.3%)	1	(8.3%)	4	(18.2%)

*UC* ulcerative colitis, *CD* Crohn’s disease, *AE* adverse event, *sAE* serious adverse event, *ADE* adverse device effect, *FP* feasibility problem, *GMA* granulocyte and monocyte adsorptive apheresis

### Summary of GMA treatment

In total, 3863 GMA sessions were administered in 437 patients (the median per patient was 10; range 1–11). Furthermore, among the 368 patients with UC, 262 (71.2%) patients received 10 sessions, 11 (3.0%) received 11 sessions and the remaining 95 (25.8%) discontinued after ≤9 sessions. In patients with CD, from the 69 patients, 34 (49.3%) received 10 GMA sessions and the remaining 35 (50.7%) had discontinued after receiving ≤9 sessions. In total, 128 patients discontinued the GMA therapy for the following reasons: 1) withdrawal of the attending physicians (*n* = 103); 2) experienced AE (*n* = 11); 3) patients own will (n = 10) and 4) withdrawal due to FP (*n* = 4). Regarding the anti-coagulant used during the preparation and administration of GMA, heparin was used in 242 patients (55.4%), nafamostat mesylate in 156 (35.7%) and low-molecular weight heparin in 33 (7.6%) at the 1st GMA session.

### Safety assessment

Overall, 437 patients were included in the safety assessment. AEs, sAEs, ADEs and FPs of all patients in each sub-group of special situations are shown in Table 1. Among all patients, AEs, sAEs, ADEs and FPs were observed in 50 (11.4%), 16 (3.7%), 11 (2.5%) and 71 (16.2%) patients, respectively. Complete information on the AEs, sAEs and ADEs are provided in Table 2. AEs in ≥1% of patients included headache in 10 patients (2.3%), nausea/vomiting in 9 (2.1%) and fever in 6 (1.4%). Similarly, sAEs included disseminated intravascular coagulation in 3 (0.7%), sepsis in 3 (0.7%), hypotension in 2 (0.5%) and pyelonephritis, febrile neutropenia, pancytopenia, aspiration pneumonitis, thromboembolic event, aortitis syndrome (Takayasu’s arteritis), atrial fibrillation and bile duct stone in 1 patient each (0.2%). However, the causality of these sAEs to GMA was ruled out by the attending physicians due to the patients’ pre-GMA serious conditions caused by diseases. Similarly, ADEs included headache in 6 patients (1.4%), nausea/vomiting in 2 (0.5%), fever in 2 (0.5%), abdominal discomfort in 2 (0.5%), peripheral neuropathy in 2 (0.5%), abnormal liver function test in 1 (0.2%) and back pain in 1 (0.2%). All these ADEs were classified as not serious. Regarding FP, blood access failure (13.5%) was the most common FP. The details on the FPs are provided in the Additional file 2: Table S2.

**Table 2:** Adverse events observed in all patients who received the GMA therapy (*n* = 437)

Observation		AE		sAE		ADE	
Total number and (%) of patients with AE, sAE, ADE		50	(11.4)	16	(3.7)	11	(2.5)
Number (%) of patients with:							
Common disorders and administration site conditions	Fever	6	(1.4)	0		2	(0.5)
	Chill	1	(0.2)	0		0	
Gastrointestinal disorder	Nausea/Vomiting	9	(2.1)	0		2	(0.5)
	Abdominal discomfort	3	(0.7)	0		2	(0.5)
Nervous system disorder	Headache	10	(2.3)	0		6	(1.4)
	Peripheral neuropathy	2	(0.5)	0		2	(0.5)
Laboratory test value abnormality	Abnormal liver function test	4	(0.9)	0		1	(0.2)
Mental disorder	Dysphoria	4	(0.9)	0		0	
	Panic disorder	1	(0.2)	0		0	
Infectious disease and parasitic disease	Sepsis	3	(0.7)	3	(0.7)	0	
	Pyelonephritis	1	(0.2)	1	(0.2)	0	
	Common cold	2	(0.5)	0		0	
	Catheter-related infection	1	(0.2)	0		0	
Blood and lymphatic system disorder	Disseminated intravascular coagulation	3	(0.7)	3	(0.7)	0	
	Febrile neutropenia	1	(0.2)	1	(0.2)	0	
	Pancytopenia	1	(0.2)	1	(0.2)	0	
Respiratory, thorax, and mediastinal disorder	Aspiration pneumonitis	1	(0.2)	1	(0.2)	0	
	Oropharyngeal discomfort	1	(0.2)	0		0	
	Hypoxia	1	(0.2)	0		0	
Musculoskeletal system and connective tissue disorder	Back pain	1	(0.2)	0		1	(0.2)
	Low back pain	1	(0.2)	0		0	
	Coxitis	1	(0.2)	0		0	
Vascular disorder	Hypotension	3	(0.7)	2	(0.5)	0	
	Thromboembolic event	1	(0.2)	1	(0.2)	0	
	Aortitis syndrome (Takayasu’s arteritis)	1	(0.2)	1	(0.2)	0	
Cardiac disorder	Palpitation	1	(0.2)	0		0	
	Atrial fibrillation	1	(0.2)	1	(0.2)	0	
Immune system disorder	Drug hypersensitivity	3	(0.7)	0		0	
Hepatobiliary disorder	Bile duct stone	1	(0.2)	1	(0.2)	0	

*GMA* granulocyte and monocyte adsorptive apheresis, *AE* adverse event, *sAE* serious adverse event, *ADE* adverse device effect

The details of the incidence of AEs, ADEs and FPs in the five major special situation sub-groups are summarised in Table 3. The incidence of AEs was the least in patients who received GMA retreatment in comparison with that in all patients and other patient sub-groups of the major special situation. In the elderly patients (age ≥ 65 years), the incidence of AEs was similar in all patients, whereas the incidences of AEs in patients on multiple immunosuppressant medications, patients with anaemia and paediatric/adolescent patients (age ≤ 18 years) were higher as compared to those in all patients and other sub-groups of the major special situation. Nausea/vomiting and headache were the most common AEs in patients on multiple immunosuppressant medications (5.6 and 3.2%, respectively), patients with anaemia (4.8 and 3.8%, respectively) and paediatric/adolescent (5.7 and 3.8%, respectively) patients. AEs observed in the five major special situation sub-groups are listed in Additional file 3: Table S3. ADE was not observed in elderly patients (Table 1). Regarding FP, the incidence of FPs was the least in patients who received GMA retreatment as compared to that in all patients and other sub-groups of the major special situation. The incidence of FPs in patients with anaemia and paediatric/adolescent were higher as compared to those in all patients and other sub-groups of the major special situation.

**Table 3:** Safety assessment of the GMA in the five major special situation sub-groups with the incidence and 95% CI

		AE			ADE			FP		
Patients/sub-group	Total	n	(%)	95% CI	n	(%)	95% CI	n	(%)	95% CI
Patients eligible for safety analysis	437	50	(11.4)		11	(2.5)		71	(16.2)	
Patients who received GMA retreatment	131	13	(9.9)	0.054–0.164	3	(2.3)	0.008–0.076	11	(8.4)	0.043–0.145
Patients on multiple immunosuppressant medications	125	19	(15.2)	0.094–0.227	5	(4.0)	0.009–0.080	23	(18.4)	0.120–0.263
Elderly patients (≥65 years)	125	14	(11.2)	0.063–0.181	0	(0.0)	0.000–0.029	22	(17.6)	0.114–0.254
Patients with anaemia (haemoglobin < 10 g/dL)	105	19	(18.1)	0.113–0.268	4	(3.8)	0.016–0.108	22	(21.0)	0.136–0.300
Paediatric/adolescent patients (≤18 years)	53	10	(18.9)	0.094–0.320	3	(5.7)	0.021–0.182	11	(20.8)	0.108–0.341

*GMA* granulocyte and monocyte adsorptive apheresis, *CI* confidence interval, *AE* adverse event, *ADE* adverse device effect, *FP* feasibility problem

**Fig. 2:**
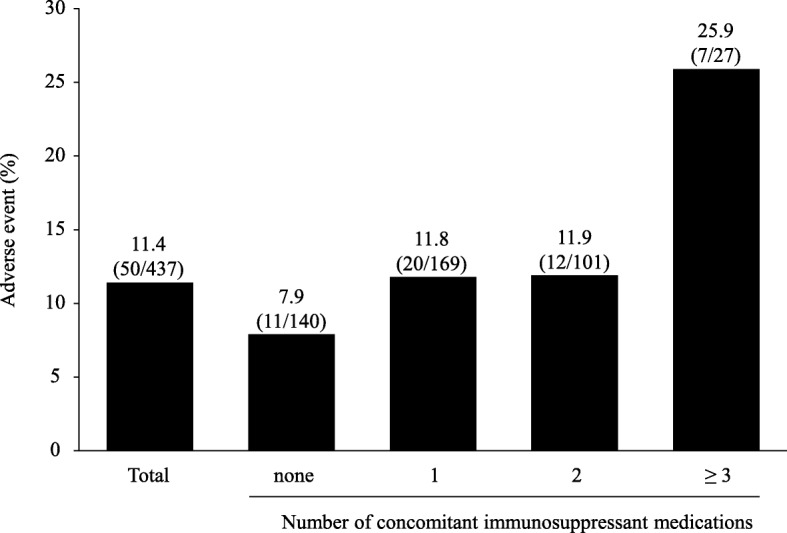
The incidences of AEs according to the number of concomitant immunosuppressant medications. *AE* adverse event

**Fig. 3:**
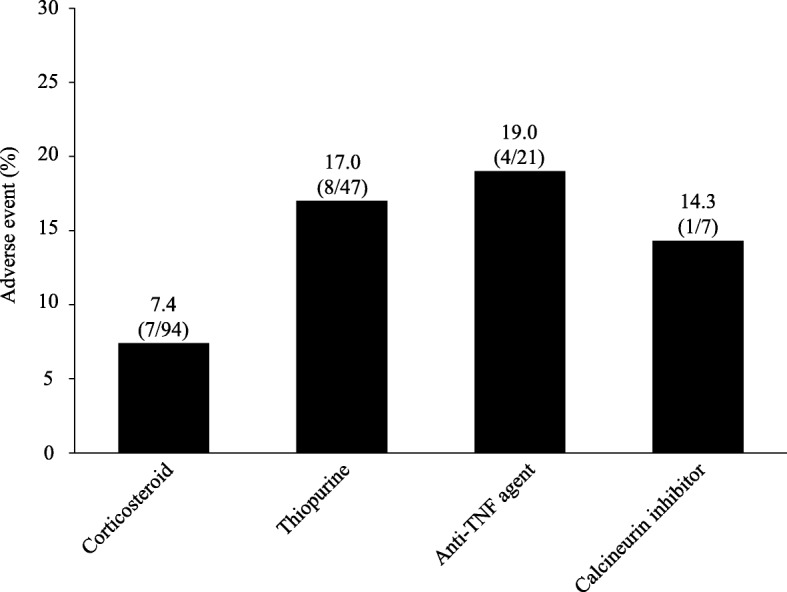
The incidences of AEs according to the type of concomitant immunosuppressant medication in patients receiving 1 concomitant medication. *AE* adverse event, *TNF* tumour necrosis factor

The incidence of AEs according to the number of concomitant immunosuppressant medications is shown in Fig. 2. A total of 140, 169, 101 and 27 patients received none, 1, 2 and ≥ 3 immunosuppressant medications, respectively. The incidence of AE was 7.9, 11.8, 11.9 and 25.9%, respectively, in patients receiving none, 1, 2 and ≥ 3 immunosuppressant medications (Fig. 2). Among patients receiving one concomitant immunosuppressant medication, 94, 47, 21 and 7 patients received corticosteroid, thiopurine, anti-TNF agent and calcineurin inhibitor, respectively. The incidence of AE was 7.4, 17.0, 19.0 and 14.3%, respectively, in patients receiving corticosteroid, thiopurine, anti-TNF agent and calcineurin inhibitor as the only concomitant immunosuppressant medication (Fig. 3). However, there was no difference between AEs observed in patients who did not receive immunosuppressant medication (7.9%) and those in patients who received corticosteroid as the only concomitant immunosuppressant medication (7.4%).

In multivariate analysis, anaemia (Hb < 10 g/dL) and concomitant immunosuppressant medications were independently associated with the incidence of AEs. Particularly, higher number of concomitant immunosuppressant medications revealed an increasing trend in the ORs related to AE (Table 4). In contrast, concomitant corticosteroid was associated with reduced risk of experiencing an AE (Table 4).

**Table 4:** Outcomes of multivariate analysis to determine risk factors for adverse event of the GMA therapy

Variable	Model 1 (forced entry)			Model 2 (backward elimination*)		
	OR	95% CI	*P* value	OR	95% CI	*P* value
Patients who received corticosteroid	0.370	0.168–0.817	0.014	0.378	0.173–0.827	0.015
Number of concomitant immunosuppressant medications						
0	Reference					
1	2.815	1.161–6.824	0.022	2.724	1.131–6.561	0.025
2	3.559	1.252–10.112	0.017	3.335	1.187–9.374	0.022
≥ 3	12.879	3.303–50.225	< 0.001	11.764	3.091–44.77	< 0.001
Paediatric/adolescent patients (≤18 years)	2.601	1.034–6.542	0.042	2.075	0.926–4.648	0.076
Elderly patients (≥65 years)	1.307	0.611–2.796	0.490			
Patients who received GMA retreatment	1.120	0.518–2.421	0.774			
Patients with anaemia (haemoglobin < 10 g/dL)	2.124	1.088–4.146	0.027	2.019	1.054–3.867	0.034
Sex (female)	1.595	0.835–3.045	0.157	1.582	0.838–2.984	0.157
Type of IBD (UC)	0.506	0.196–1.308	0.160	0.519	0.204–1.322	0.169
Duration of disease (year)	1.015	0.978–1.054	0.434			

*GMA* granulocyte and monocyte adsorptive apheresis, *IBD* inflammatory bowel disease, *UC* ulcerative colitis, *OR* odds ratio, *CI* confidence interval

*Variables with *P* > 0.2 were excluded via the backward elimination method

### Effectiveness of the GMA treatment

**Fig. 4:**
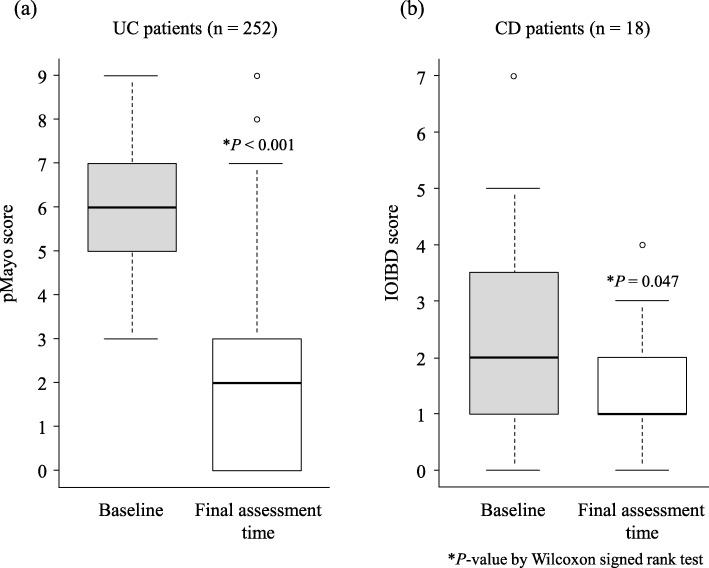
Comparison of median pMayo score at baseline and at the final assessment time in UC (**a**) and IOIBD score in CD (**b**). *UC* ulcerative colitis, *CD* Crohn’s disease, *pMayo* partial Mayo, *IOIBD* International Organization for the Study of Inflammatory Bowel Diseases

**Fig. 5:**
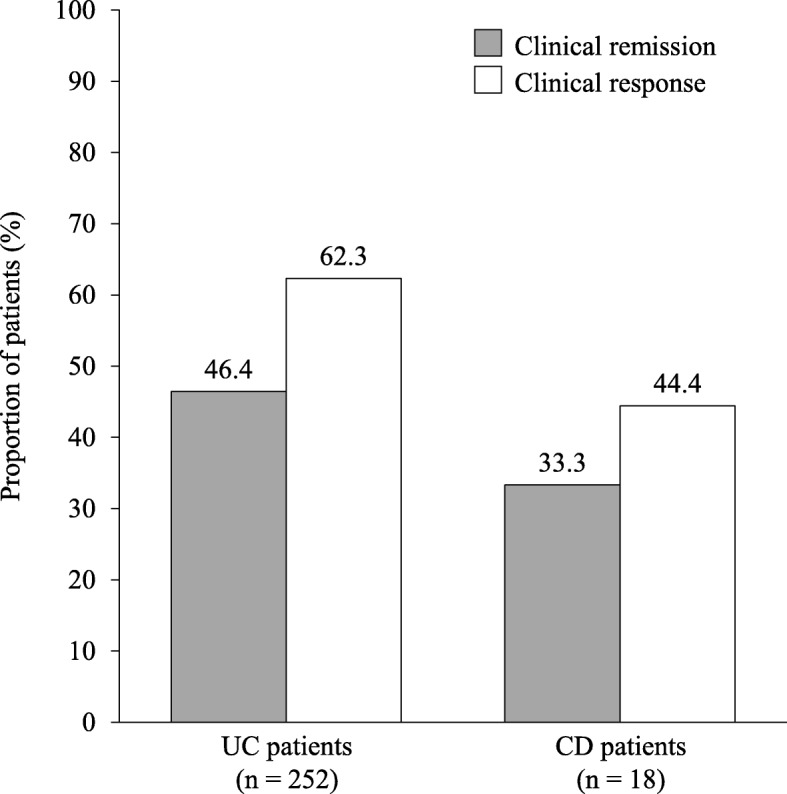
Clinical remission and response rates of the GMA therapy in patients with UC and CD in special situation. *CD* Crohn’s disease, G*MA* granulocyte and monocyte adsorptive apheresis, *UC* ulcerative colitis

**Fig. 6:**
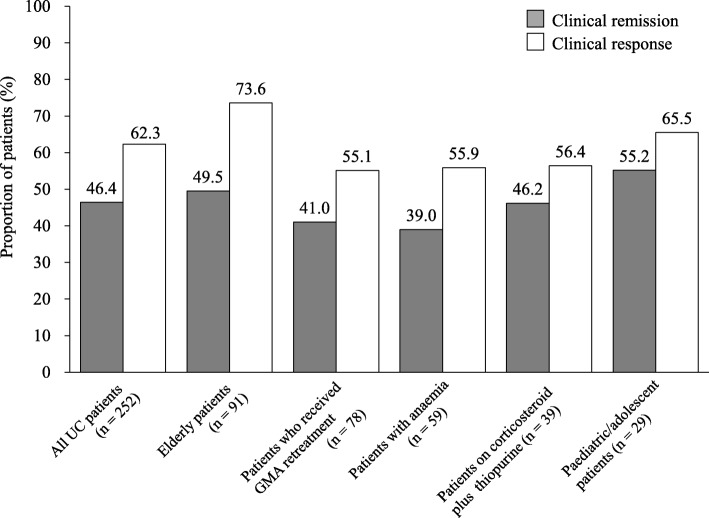
Clinical remission and response rates of the GMA therapy in the five major special situation sub-groups of patients with UC. *UC* ulcerative colitis, *GMA* granulocyte and monocyte adsorptive apheresis

In the effectiveness assessment, 116 patients with UC and 51 patients with CD did not meet the inclusion criteria for effectiveness assessment. Therefore, 252 patients with UC and 18 patients with CD were eligible for effectiveness assessment. The pMayo and IOIBD scores at the final assessment time were significantly decreased compared with the baseline scores (Fig. 4). In patients with UC, the median pMayo scores at baseline and at the final assessment time were 6 (IQR 5–7) and 2 (IQR 0–3), respectively (*P* < 0.001), whereas in patients with CD, the median IOIBD scores at baseline and at the final assessment time were 2 (IQR 1–3.5) and 1 (IQR 1–2), respectively (*P* = 0.047). Figure 5 shows clinical remission and response rates for all patients who were included in the analysis for the effectiveness of GMA treatment. Among the 252 patients with UC, 117 (46.4%) and 161 (62.3%) achieved clinical remission and clinical response, respectively. Of these patients, 91 were elderly patients (remission 49.5%, response 73.6%), 78 had received GMA retreatment (remission 41.0%, response 55.1%), 59 were anaemic (remission 39.0%, response 55.9%), 39 were on corticosteroid plus thiopurine (remission 46.2%, response 56.4%) and 29 were paediatric/adolescent patients (remission 55.2%, response 65.5%, Fig. 6). Additional information on the effectiveness of GMA in the five major special situation sub-groups of UC is provided in Additional file 4: Figure S1. Of the 18 patients with CD, 6 (33.3%) and 8 (44.4%) patients achieved clinical remission and clinical response, respectively (Fig. 5).

### Corticosteroid-sparing effect of GMA

**Fig. 7:**
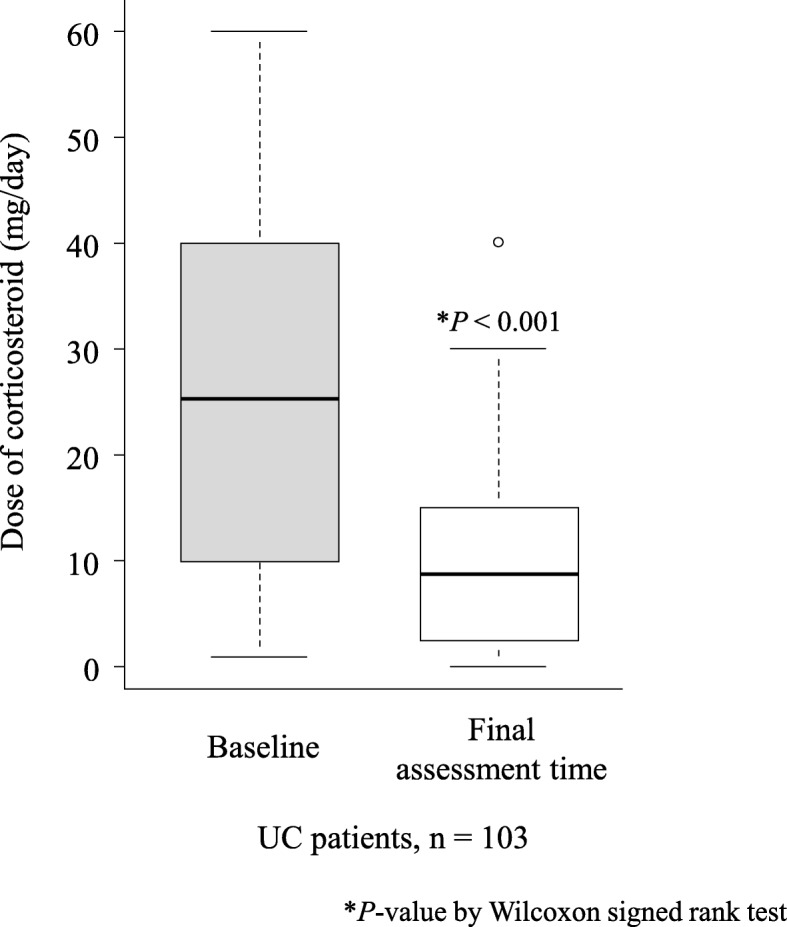
Comparison of median dose of corticosteroid at baseline and at the final assessment time in patients with UC (*n* = 103) who were on corticosteroid at baseline. *UC* ulcerative colitis

At baseline, 103 patients with UC received corticosteroid. Figure 7 shows the changes in corticosteroid dose in patients with UC determined at baseline and at the final assessment time. The median dose of corticosteroid was significantly reduced from 25 (IQR 10–40) mg/day at baseline to 10 (IQR 3.75–15) mg/day at the final assessment time (P < 0.001). Among the 103 patients with UC who received corticosteroid at baseline, 22 (21.4%) had discontinued corticosteroid treatment and 86 (83.5%) had tapered the corticosteroid dose at the final assessment time.

## Discussion

To the best of our knowledge, this is the first retrospective, multicentre cohort study that sought to assess the safety and effectiveness of GMA therapy in patients with IBD under special situations. For the safety analysis, 437 eligible patients (368 UC, 69 CD) were analysed, and we focused on the evaluation of AEs and FPs related to GMA treatment. For the analysis of effectiveness, a total of 270 eligible patients (252 UC, 18 CD) were assessed. Furthermore, among the patients, 131 patients received GMA retreatment, 125 of them received concomitant multiple immunosuppressant medications, 125 were elderly patients, 105 were patients with anaemia and 53 were paediatrics/adolescents and other smaller sub-groups.

The overall incidence of AEs in our safety analysis was 11.4%, which is > 7.7% as per the report by Hibi et al. [26] in an earlier retrospective study involving patients treated with GMA in routine clinical settings. This result may be explained, at least partially, by the fact that from 1999 to 2006, when Hibi et al. conducted their study, thiopurines, calcineurin inhibitors and anti-TNF agents were off-label in Japan. The rate of concomitant thiopurine administration in a previous study was only 9.8% [26]. Conversely, in the present study, thiopurines, calcineurin inhibitors and anti-TNF agents were administrated in 30, 4.6 and 20.4% patients, respectively. This finding may indicate that some of our patients developed more complicated or severe diseases than that in patients of the previous study [26]. Similarly, the AE rate for GMA reported in a cohort of patients with IBD in Scandinavia was 15%, with 44% of UC and 37% of CD on concomitant thiopurines and 66% of patients with CD who had previously received anti-TNF agents [31]. Similarly, the AE rate for GMA reported in a cohort of patients with UC in Spain was 18% with 68% on concomitant thiopurines and 16.2% with a previous exposure to anti-TNF agents [32]. Furthermore, with a filter-type leukocytapheresis (LCAP) system, Yokoyama et al. [46] reported an AE rate of 10.3% in regular patients with UC, which is close to the rate obtained in the present study. Here, thiopurines, calcineurin inhibitors and anti-TNF agents were administrated in 32.8, 12.3 and 1.1% of the patients, respectively. Considering that the rate of concomitant use of immunosuppressant medications and the incidence of AE in the present study were almost the same as in past cohort studies [31, 32, 46], the concomitant use of multiple immunosuppressant medication may have contributed to accelerated AEs, which resulted in higher numbers than those reported by Hibi et al. [26]. The fact that the concomitant use of multiple immunosuppressant medications was the prognostic factor that contributed to an increase of AEs in the present study also supported the notion.

The incidences of AE in patients receiving none, 1, 2 and ≥ 3 immunosuppressant medications were 7.9, 11.8, 11.9 and 25.9%, respectively. As the number of concomitant immunosuppressant medications increased, the incidence of AEs also increased. Furthermore, in the multivariate analysis, a higher number of concomitant immunosuppressant medications showed an increasing trend in the ORs related to AE, and we found that concomitant corticosteroids was associated with a reduced risk of AE. Considering that patients with special situations were included in this study, the AE rate in patients on concomitant corticosteroids was 7.4%, which is almost the same as that reported in the study by Hibi et al. (7.7%) but lower than that for the total population in this study (11.4%). Therefore, it would appear that corticosteroids might not reduce the risk of AE.

We also found that anaemia (Hb < 10 g/dL) was an independent prognostic factor that contributed to the incidence of AE in the present study. The present study was the first to report an association between anaemia and the incidence of AEs for GMA. Presently, the mechanism(s) by which anaemia increases AE incidence remains unclear.

In the present study, we found that concomitant multiple immunosuppressant medications and anaemia were associated with higher incidence of AE, including nausea/vomiting (5.6 and 3.2%) and headache (4.8 and 3.8%), which were higher than the rate of nausea (0.86%) and headache (1.58%) reported by Hibi, et al. [26]. However, only two patients discontinued GMA therapy due to nausea/vomiting in both these sub-groups, and the response rate was > 50%, which indicated that GMA therapy should not be hesitated in patients with multiple immunosuppressant medications or anaemia (data not presented).

Regarding the effectiveness of GMA in the present study, the remission rate in patients with UC (46.4%) was lower than that reported by Hibi et al. (71.1%) [26]. On the other hand, the remission rates in the aforementioned Scandinavian and Spanish studies were reported to be 40 and 37%, respectively, which are close to our results. In the present study, patients who received additional pharmacological therapy while undertaking GMA therapy were considered as GMA non-responders. The Scandinavian study also considered patients who needed an increased dose of corticosteroid as non-responders to GMA. Such criteria were not considered in the Hibi et al.’s study [26], and might explain the different remission rates between the two studies. In the present study, in the five major special situations with UC, we observed that clinical remission was achieved in > 40% of patients and GMA therapy appeared to be effective regardless of the patients’ special situations. Thus, it can be inferred that GMA could be a relevant therapeutic option in these patient sub-groups.

In this report, we have described the assessment of the safety and effectiveness of GMA in 125 elderly patients [age ≥ 65 years] with IBD, which is the largest number ever reported for an extracorporeal therapy for IBD. In the elderly group, the AE rate was not high (11.2%), in fact, it was nearly the same as the average for the total population, and the remission rate for elderly patients with UC was 49.5%, which was slightly higher as compared with the average rate of 46.4% for all eligible patients. Komoto et al. [47] reported on the safety and effectiveness of LCAP in 75 regular elderly patients with UC who were assessed in the same period of our study. More recently, Ito et al. [48] described the safety and effectiveness of GMA in a small cohort of 24 regular elderly patients with UC. The remission and AE rates reported by the authors were 70.8–78.5% and 8.0–12.0%, respectively. The AE rates in the two aforementioned studies were reported to be extremely close to the rate obtained in the present study [47, 48]. Therefore, GMA promises to have a reasonably good safety for use in elderly patients with IBD.

Furthermore, adolescent patients/adolescent patients (age ≤ 18 years), an 18.9% AE rate did not appear to be significantly high in multivariate analysis. With regard to the safety of GMA in the paediatric setting, in a previous multicentre cohort study by Ruuska et al. [49], the AE rate was reported to be 32%, which is much higher than that in our study, while in another multicentre cohort study by Tomomasa et al. [50], the AE rate was 9.1%, which is almost half of the rate in the present GMA study. However, notably, the safety reports for GMA in paediatric setting were less than that in adult settings; therefore, a comprehensive comparison with the published studies was not possible at this time-point. Nonetheless, the present study revealed that the safety and effectiveness for GMA in paediatric patients were similar to those for all patients, which includes mostly adult patients with IBD. Therefore, GMA should be a relevant treatment option in paediatric patients.

The strength of our study includes a relatively large number of patients who are difficult to evaluate in clinical trials or in routine practice settings; however, the present study has several limitations. First, because of the retrospective nature of the study, the incidence of AEs may have been underestimated. Second, since we focused on patients with at least one special situation [6–10], a comparison with the outcomes in patients with IBD without special situation was not possible. In addition, because of the overlapping special situation in patients, a comparative analysis among different special situation sub-groups was also insufficient. Furthermore, other smaller special situation sub-groups, including pregnant patients, could not be evaluated in detail. However, since the present study was the first cohort to evaluate the safety and effectiveness of GMA treatment in patients with IBD with special situations, we believe that the results of the PARTICULAR study provide information that would be important for the treatment of patients with IBD with special situations.

Finally, the nature of an observational study in a real-world clinical practice setting, with the absence of any limitation on concomitant medications potentially may lead to a bias in the assessment of effectiveness. In addition, the evaluation of effectiveness did not include endoscopic findings and faecal calprotectin to support the clinical assessment of GMA effectiveness. Furthermore, in this study, we decided to exclude patients who were receiving an anti-TNF agent to better understand the full effectiveness of GMA in these patients. Therefore, further prospective studies are warranted to fully evaluate the effectiveness of GMA in special situations as well as patients on biologics.

## Conclusion

We conducted the first retrospective, multicentre cohort study to evaluate the safety and effectiveness of GMA therapy in patients with IBD under special situations. The overall incidence of AEs and the overall remission rate in patients with UC were similar to that previously reported for GMA in studies of regular patients within the European countries. Particularly, the incidence of AEs in the elderly patients was not higher than that of all patients. Therefore, GMA can be considered as a safe treatment option in elderly patients with IBD. In contrast, the incidences of AEs were higher in patients with anaemia and in patients on multiple concomitant immunosuppressant medications. During GMA treatment in these patients, extra care should be taken for AE such as nausea/vomiting and headache. To further strengthen the clinical relevance of the outcomes in the present study, a prospective study with a larger number of patients with special situations is warranted.

## Supplementary information

**Additional file 1 :** **Table S1** Baseline demographic variables of the eligible UC and CD patients for inclusion in this study.

**Additional file 2 : Table S2** Feasibility problems observed in all patients who received the GMA therapy (*n* = 437).

**Additional file 3 : Table S3** AE observed in safety assessment of the GMA therapy in the five major special situation sub-groups.

**Additional file 4 : Figure S1** Comparison of pMayo scores at baseline and at the final assessment time in the five major special situation sub-groups of patients with UC.

## Data Availability

The datasets used during the current study are available from the corresponding author upon reasonable request.

## Abbreviations

CD: Crohn’s disease
GMA: Granulocyte and monocyte adsorptive apheresis
IBD: Inflammatory bowel disease
IOIBD: International Organization for the Study of Inflammatory Bowel Diseases
pMayo: partial Mayo
TNF: Tumour necrosis factor
UC: Ulcerative colitis
