# Supplementary material for: Safety and effectiveness of granulocyte and monocyte adsorptive apheresis in patients with inflammatory bowel disease in special situations: a multicentre cohort study
Source: BMC Gastroenterol. 2019 Nov 21;19:196. doi: 10.1186/s12876-019-1110-1 (PMC6873503; doi:10.1186/s12876-019-1110-1)
Supplement: Supplementary file 1 — Additional file 1 : Table S1 Baseline demographic variables of the eligible UC and CD patients for inclusion in this study. [file 12876_2019_1110_MOESM1_ESM.pptx]

## Slide 1
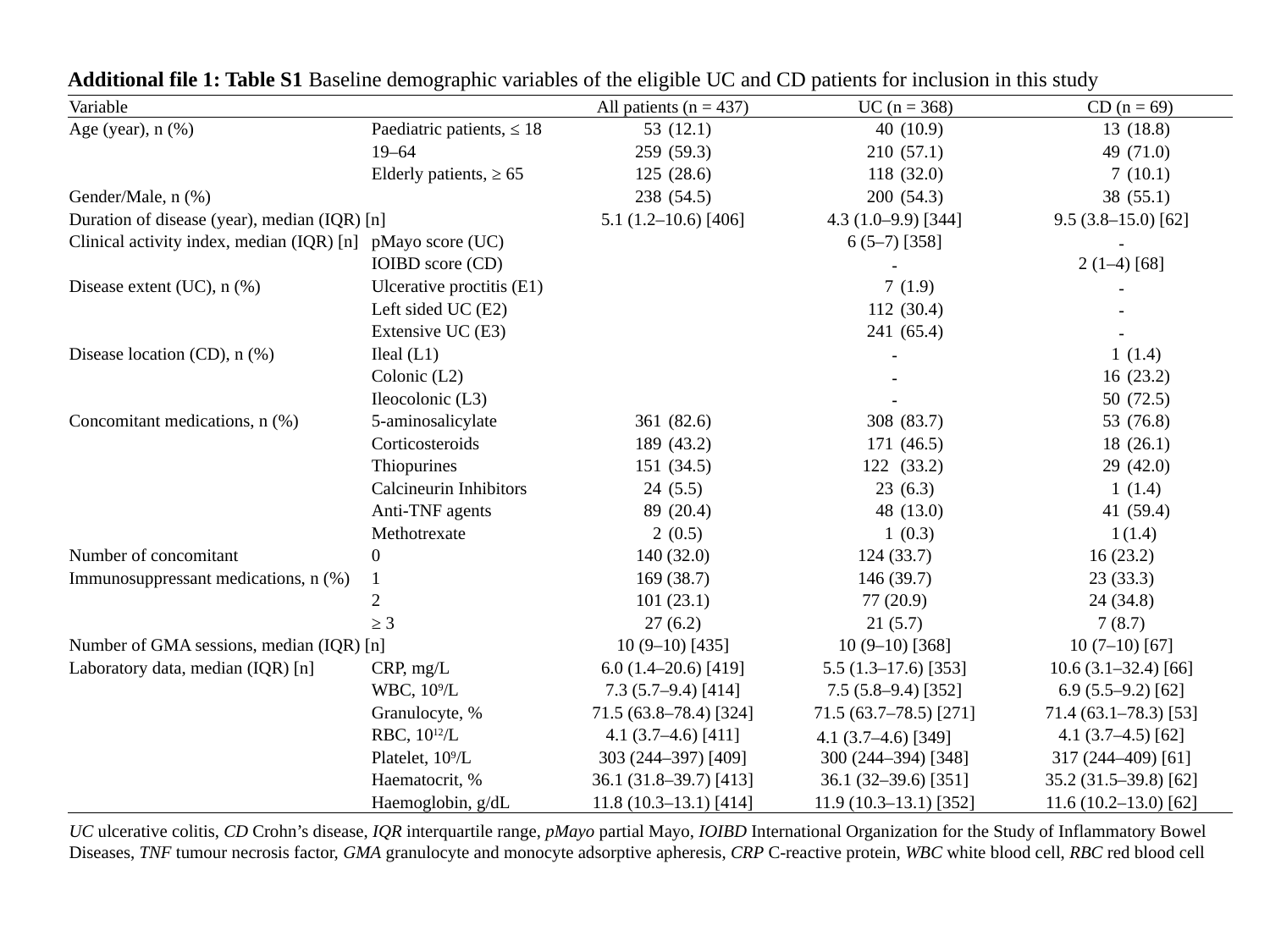

Additional file 1: Table S1 Baseline demographic variables of the eligible UC and CD patients for inclusion in this study
| Variable | | All patients (n = 437) | | UC (n = 368) | | CD (n = 69) | |
| --- | --- | --- | --- | --- | --- | --- | --- |
| Age (year), n (%) | Paediatric patients, ≤ 18 | 53 | (12.1) | 40 | (10.9) | 13 | (18.8) |
| | 19–64 | 259 | (59.3) | 210 | (57.1) | 49 | (71.0) |
| | Elderly patients, ≥ 65 | 125 | (28.6) | 118 | (32.0) | 7 | (10.1) |
| Gender/Male, n (%) | | 238 | (54.5) | 200 | (54.3) | 38 | (55.1) |
| Duration of disease (year), median (IQR) [n] | | 5.1 (1.2–10.6) [406] | | 4.3 (1.0–9.9) [344] | | 9.5 (3.8–15.0) [62] | |
| Clinical activity index, median (IQR) [n] | pMayo score (UC) | | | 6 (5–7) [358] | | - | |
| | IOIBD score (CD) | | | - | | 2 (1–4) [68] | |
| Disease extent (UC), n (%) | Ulcerative proctitis (E1) | | | 7 | (1.9) | - | |
| | Left sided UC (E2) | | | 112 | (30.4) | - | |
| | Extensive UC (E3) | | | 241 | (65.4) | - | |
| Disease location (CD), n (%) | Ileal (L1) | | | - | | 1 | (1.4) |
| | Colonic (L2) | | | - | | 16 | (23.2) |
| | Ileocolonic (L3) | | | - | | 50 | (72.5) |
| Concomitant medications, n (%) | 5-aminosalicylate | 361 | (82.6) | 308 | (83.7) | 53 | (76.8) |
| | Corticosteroids | 189 | (43.2) | 171 | (46.5) | 18 | (26.1) |
| | Thiopurines | 151 | (34.5) | 122 | (33.2) | 29 | (42.0) |
| | Calcineurin Inhibitors | 24 | (5.5) | 23 | (6.3) | 1 | (1.4) |
| | Anti-TNF agents | 89 | (20.4) | 48 | (13.0) | 41 | (59.4) |
| | Methotrexate | 2 | (0.5) | 1 | (0.3) | 1 | (1.4) |
| Number of concomitant | 0 | 140 (32.0) | | 124 (33.7) | | 16 (23.2) | |
| Immunosuppressant medications, n (%) | 1 | 169 (38.7) | | 146 (39.7) | | 23 (33.3) | |
| | 2 | 101 (23.1) | | 77 (20.9) | | 24 (34.8) | |
| | ≥ 3 | 27 (6.2) | | 21 (5.7) | | 7 (8.7) | |
| Number of GMA sessions, median (IQR) [n] | | 10 (9–10) [435] | | 10 (9–10) [368] | | 10 (7–10) [67] | |
| Laboratory data, median (IQR) [n] | CRP, mg/L | 6.0 (1.4–20.6) [419] | | 5.5 (1.3–17.6) [353] | | 10.6 (3.1–32.4) [66] | |
| | WBC, 109/L | 7.3 (5.7–9.4) [414] | | 7.5 (5.8–9.4) [352] | | 6.9 (5.5–9.2) [62] | |
| | Granulocyte, % | 71.5 (63.8–78.4) [324] | | 71.5 (63.7–78.5) [271] | | 71.4 (63.1–78.3) [53] | |
| | RBC, 1012/L | 4.1 (3.7–4.6) [411] | | 4.1 (3.7–4.6) [349] | | 4.1 (3.7–4.5) [62] | |
| | Platelet, 109/L | 303 (244–397) [409] | | 300 (244–394) [348] | | 317 (244–409) [61] | |
| | Haematocrit, % | 36.1 (31.8–39.7) [413] | | 36.1 (32–39.6) [351] | | 35.2 (31.5–39.8) [62] | |
| | Haemoglobin, g/dL | 11.8 (10.3–13.1) [414] | | 11.9 (10.3–13.1) [352] | | 11.6 (10.2–13.0) [62] | |
UC ulcerative colitis, CD Crohn’s disease, IQR interquartile range, pMayo partial Mayo, IOIBD International Organization for the Study of Inflammatory Bowel Diseases, TNF tumour necrosis factor, GMA granulocyte and monocyte adsorptive apheresis, CRP C-reactive protein, WBC white blood cell, RBC red blood cell
