# Supplementary material for: Safety and effectiveness of granulocyte and monocyte adsorptive apheresis in patients with inflammatory bowel disease in special situations: a multicentre cohort study
Source: BMC Gastroenterol. 2019 Nov 21;19:196. doi: 10.1186/s12876-019-1110-1 (PMC6873503; doi:10.1186/s12876-019-1110-1)
Supplement: Supplementary file 2 — Additional file 2 : Table S2 Feasibility problems observed in all patients who received the GMA therapy (n = 437). [file 12876_2019_1110_MOESM2_ESM.pptx]

## Slide 1
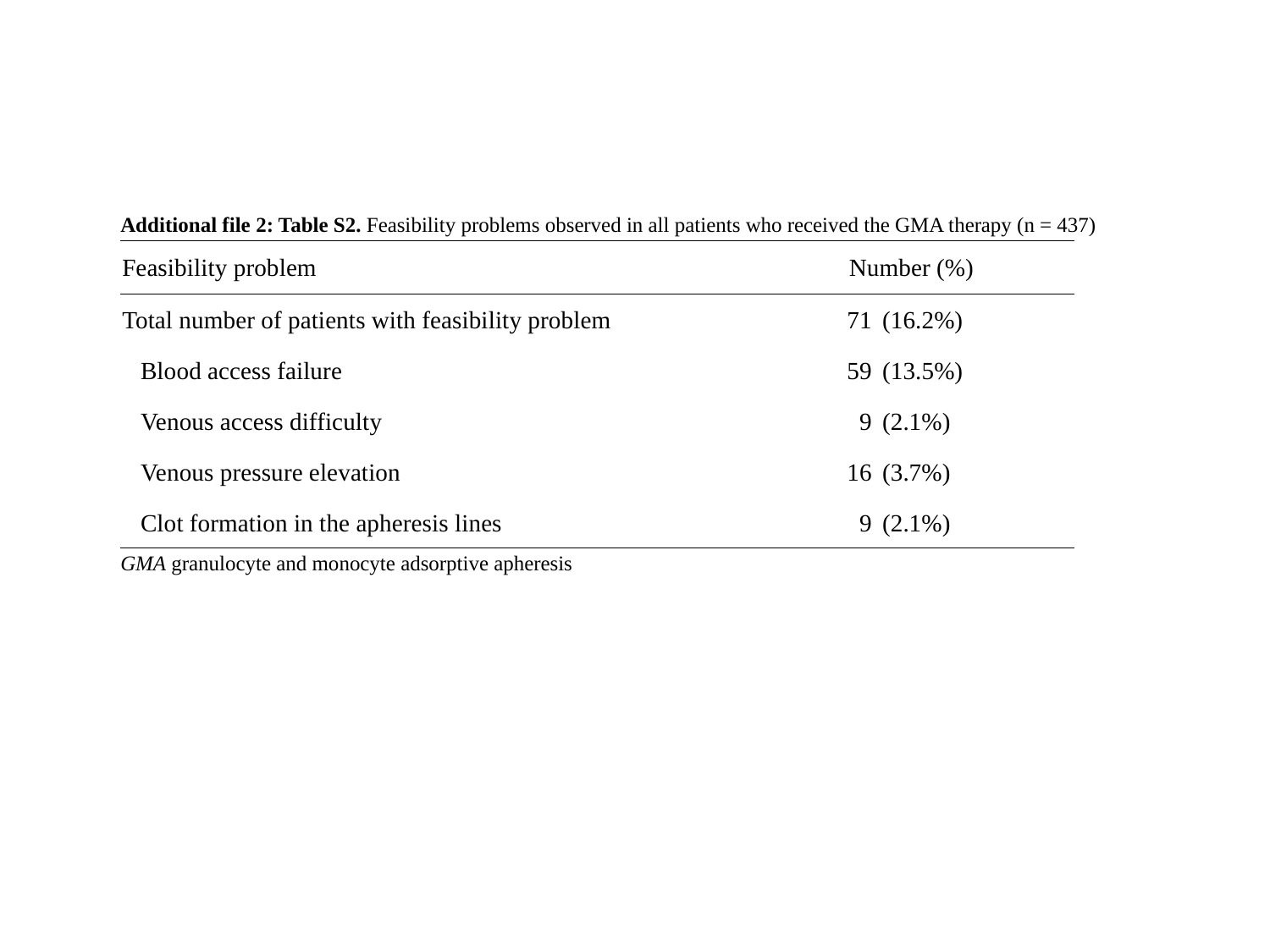

Additional file 2: Table S2. Feasibility problems observed in all patients who received the GMA therapy (n = 437)
| Feasibility problem | Number (%) | |
| --- | --- | --- |
| Total number of patients with feasibility problem | 71 | (16.2%) |
| Blood access failure | 59 | (13.5%) |
| Venous access difficulty | 9 | (2.1%) |
| Venous pressure elevation | 16 | (3.7%) |
| Clot formation in the apheresis lines | 9 | (2.1%) |
GMA granulocyte and monocyte adsorptive apheresis
