# Supplementary material for: Safety and effectiveness of granulocyte and monocyte adsorptive apheresis in patients with inflammatory bowel disease in special situations: a multicentre cohort study
Source: BMC Gastroenterol. 2019 Nov 21;19:196. doi: 10.1186/s12876-019-1110-1 (PMC6873503; doi:10.1186/s12876-019-1110-1)
Supplement: Supplementary file 3 — Additional file 3 : Table S3 AE observed in safety assessment of the GMA therapy in the five major special situation sub-groups. [file 12876_2019_1110_MOESM3_ESM.pptx]

## Slide 1
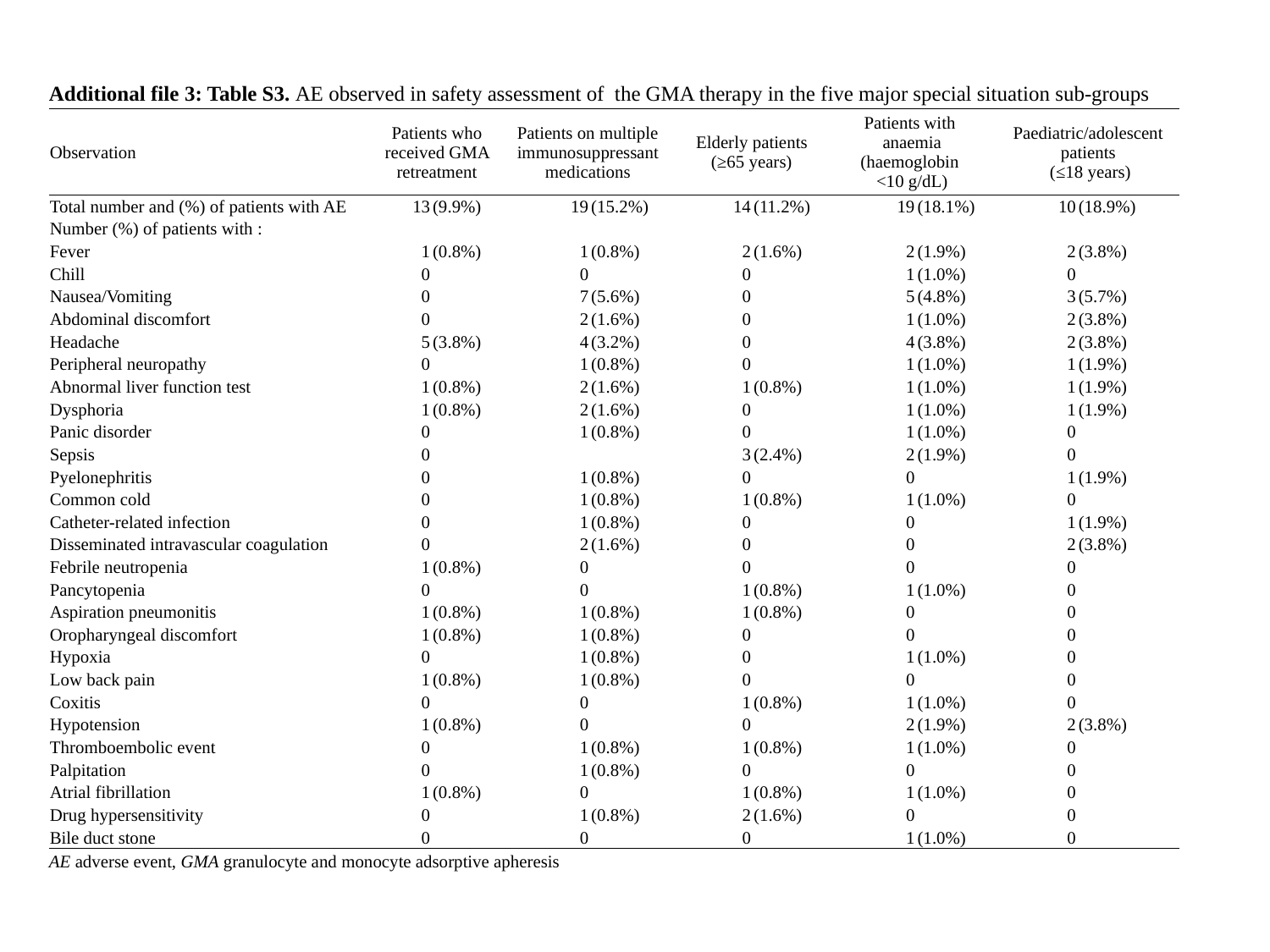

Additional file 3: Table S3. AE observed in safety assessment of the GMA therapy in the five major special situation sub-groups
| Observation | Patients who received GMA retreatment | | Patients on multiple immunosuppressant medications | | Elderly patients (≥65 years) | | Patients with anaemia (haemoglobin <10 g/dL) | | Paediatric/adolescent patients (≤18 years) | |
| --- | --- | --- | --- | --- | --- | --- | --- | --- | --- | --- |
| Total number and (%) of patients with AE | 13 | (9.9%) | 19 | (15.2%) | 14 | (11.2%) | 19 | (18.1%) | 10 | (18.9%) |
| Number (%) of patients with : | | | | | | | | | | |
| Fever | 1 | (0.8%) | 1 | (0.8%) | 2 | (1.6%) | 2 | (1.9%) | 2 | (3.8%) |
| Chill | 0 | | 0 | | 0 | | 1 | (1.0%) | 0 | |
| Nausea/Vomiting | 0 | | 7 | (5.6%) | 0 | | 5 | (4.8%) | 3 | (5.7%) |
| Abdominal discomfort | 0 | | 2 | (1.6%) | 0 | | 1 | (1.0%) | 2 | (3.8%) |
| Headache | 5 | (3.8%) | 4 | (3.2%) | 0 | | 4 | (3.8%) | 2 | (3.8%) |
| Peripheral neuropathy | 0 | | 1 | (0.8%) | 0 | | 1 | (1.0%) | 1 | (1.9%) |
| Abnormal liver function test | 1 | (0.8%) | 2 | (1.6%) | 1 | (0.8%) | 1 | (1.0%) | 1 | (1.9%) |
| Dysphoria | 1 | (0.8%) | 2 | (1.6%) | 0 | | 1 | (1.0%) | 1 | (1.9%) |
| Panic disorder | 0 | | 1 | (0.8%) | 0 | | 1 | (1.0%) | 0 | |
| Sepsis | 0 | | | | 3 | (2.4%) | 2 | (1.9%) | 0 | |
| Pyelonephritis | 0 | | 1 | (0.8%) | 0 | | 0 | | 1 | (1.9%) |
| Common cold | 0 | | 1 | (0.8%) | 1 | (0.8%) | 1 | (1.0%) | 0 | |
| Catheter-related infection | 0 | | 1 | (0.8%) | 0 | | 0 | | 1 | (1.9%) |
| Disseminated intravascular coagulation | 0 | | 2 | (1.6%) | 0 | | 0 | | 2 | (3.8%) |
| Febrile neutropenia | 1 | (0.8%) | 0 | | 0 | | 0 | | 0 | |
| Pancytopenia | 0 | | 0 | | 1 | (0.8%) | 1 | (1.0%) | 0 | |
| Aspiration pneumonitis | 1 | (0.8%) | 1 | (0.8%) | 1 | (0.8%) | 0 | | 0 | |
| Oropharyngeal discomfort | 1 | (0.8%) | 1 | (0.8%) | 0 | | 0 | | 0 | |
| Hypoxia | 0 | | 1 | (0.8%) | 0 | | 1 | (1.0%) | 0 | |
| Low back pain | 1 | (0.8%) | 1 | (0.8%) | 0 | | 0 | | 0 | |
| Coxitis | 0 | | 0 | | 1 | (0.8%) | 1 | (1.0%) | 0 | |
| Hypotension | 1 | (0.8%) | 0 | | 0 | | 2 | (1.9%) | 2 | (3.8%) |
| Thromboembolic event | 0 | | 1 | (0.8%) | 1 | (0.8%) | 1 | (1.0%) | 0 | |
| Palpitation | 0 | | 1 | (0.8%) | 0 | | 0 | | 0 | |
| Atrial fibrillation | 1 | (0.8%) | 0 | | 1 | (0.8%) | 1 | (1.0%) | 0 | |
| Drug hypersensitivity | 0 | | 1 | (0.8%) | 2 | (1.6%) | 0 | | 0 | |
| Bile duct stone | 0 | | 0 | | 0 | | 1 | (1.0%) | 0 | |
AE adverse event, GMA granulocyte and monocyte adsorptive apheresis
