# Supplementary material for: Safety and effectiveness of granulocyte and monocyte adsorptive apheresis in patients with inflammatory bowel disease in special situations: a multicentre cohort study
Source: BMC Gastroenterol. 2019 Nov 21;19:196. doi: 10.1186/s12876-019-1110-1 (PMC6873503; doi:10.1186/s12876-019-1110-1)
Supplement: Supplementary file 4 — Additional file 4 : Figure S1 Comparison of pMayo scores at baseline and at the final assessment time in the five major special situation sub-groups of patients with UC. [file 12876_2019_1110_MOESM4_ESM.pptx]

## Slide 1
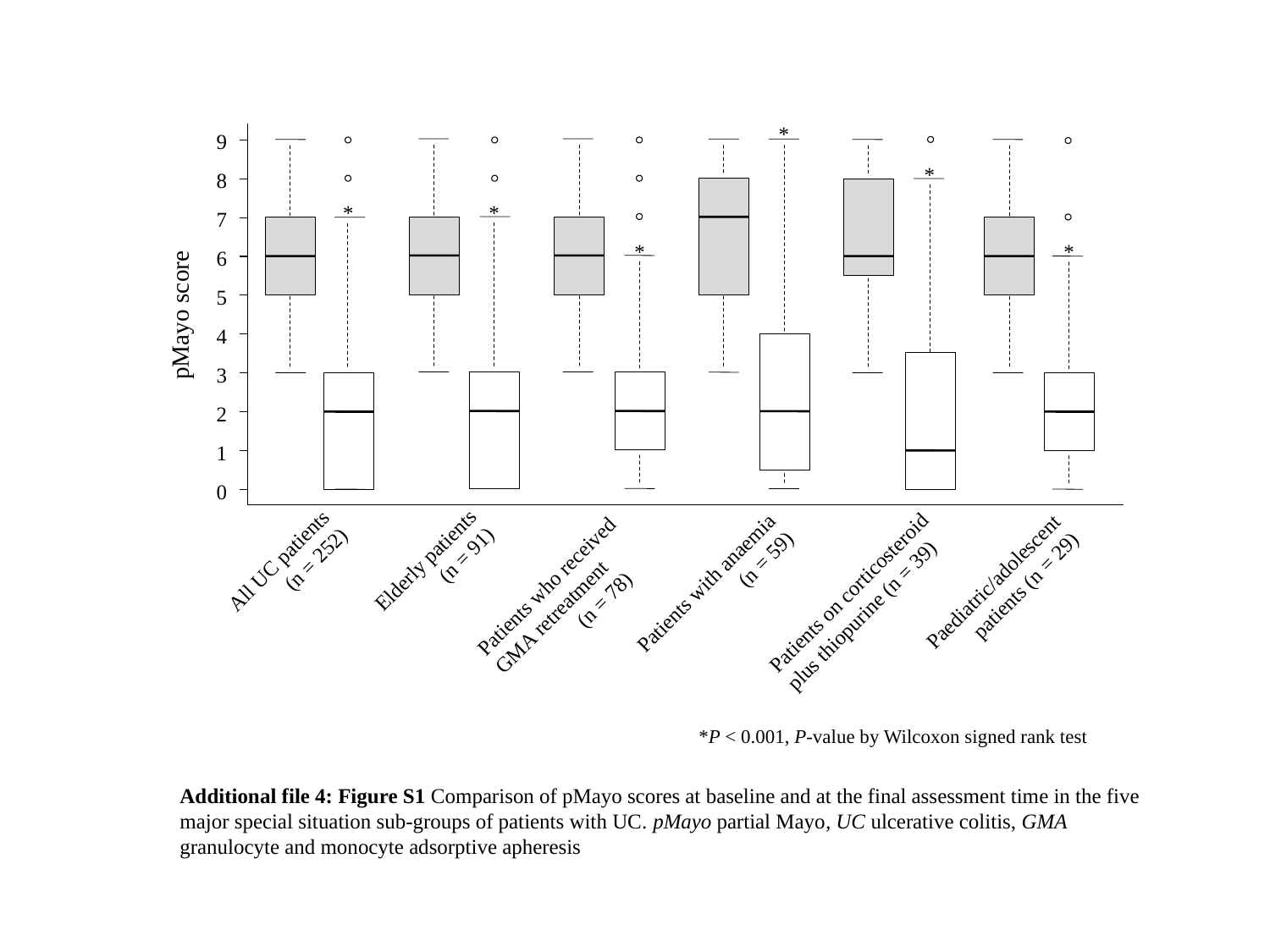

*
9
8
7
6
5
4
3
2
1
0
pMayo score
Elderly patients
 (n = 91)
All UC patients
 (n = 252)
Patients who received
GMA retreatment
 (n = 78)
Paediatric/adolescent
patients (n = 29)
Patients with anaemia
(n = 59)
Patients on corticosteroid
plus thiopurine (n = 39)
*
*
*
*
*
*P < 0.001, P-value by Wilcoxon signed rank test
Additional file 4: Figure S1 Comparison of pMayo scores at baseline and at the final assessment time in the five major special situation sub-groups of patients with UC. pMayo partial Mayo, UC ulcerative colitis, GMA granulocyte and monocyte adsorptive apheresis
